# Virological characteristics of cases of COVID-19 in northern Viet Nam,  January–May 2020

**DOI:** 10.5365/wpsar.2021.12.4.833

**Published:** 2021-12-22

**Authors:** Hang Khanh Le Nguyen, Son Vu Nguyen, Phuong Mai Vu Hoang, Thanh thi Le, Huong thi Thu Tran, Long Hai Pham Nguyen, Thai Quang Pham, Thuy thanh Nguyen, Anh Duc Dang, Anh Phuong Nguyen, Mai thi Quynh Le

**Affiliations:** a National Institute of Hygiene and Epidemiology, Hanoi, Viet Nam.; b Mohawk College, Hamilton, Ontario, Canada.

## Abstract

**Background:**

Viet Nam confirmed its first case of severe acute respiratory syndrome coronavirus 2 (SARS-CoV-2) infection on 23 January 2020 among travellers from Wuhan, China, and experienced several clusters of community transmission until September. Viet Nam implemented an aggressive testing, isolation, contact tracing and quarantine strategy in response to all laboratory-confirmed cases. We report the results of SARS-CoV-2 testing during the first half of 2020 in northern Viet Nam.

**Methods:**

Between January and May 2020, 15 650 upper respiratory tract specimens were collected from 14 470 suspected cases and contacts in northern Viet Nam. All were tested for SARS-CoV-2 by real-time RT–PCR. Individuals with positive specimens were tested every three days until two tests were negative. Positive specimens from 81 individuals were cultured.

**Results:**

Among 14 470 tested individuals, 158 (1.1%) cases of SARS-CoV-2 infection were confirmed; 89 were imported and 69 were associated with community transmission. Most patients (122, 77%) had negative results after two tests, while 11 and 4 still tested positive when sampled a third and fourth time, respectively. SARS-CoV-2 was isolated from 29 of 81 specimens (36%) with a cycle threshold (Ct) value < 30. Seven patients who tested positive again after testing negative had Ct values > 30 and negative cultures.

**Conclusion:**

Early, widespread testing for SARS-CoV-2 in northern Viet Nam identified very few cases, which, when combined with other aggressive strategies, may have dramatically contained the epidemic. We observed rapid viral clearance and very few positive results after clearance. Large-scale molecular diagnostic testing is a critical part of early detection and containment of COVID-19 in Viet Nam and will remain necessary until vaccination is widely implemented.

Severe acute respiratory syndrome coronavirus 2 (SARS-CoV-2) is the cause of coronavirus disease 2019 (COVID-19), which was first reported in Wuhan, China, in late December 2019. As of September 2020, SARS-CoV-2 was responsible for over 25 million cases and nearly 1 million deaths. (*1*)

Viet Nam is a country of 97 million people, which, despite its lower- to middle-income status, has managed to limit the spread of SARS-CoV-2, requiring 8 months to reach 1000 cases and 7 months to record its first fatality. Strategies for prevention, detection and control have included the key response measures of early detection, testing and treatment, required for all persons entering the country from affected countries, starting in early February 2020. (*2*) The early days of the pandemic in Viet Nam were marked primarily by cases imported from China, whereas the second cluster was characterized by cases mainly imported from Europe. (*2*-*6*)

Viet Nam hosts two national influenza centres, including one at the National Institute of Hygiene and Epidemiology (NIHE). The Institute coordinates influenza surveillance in northern Viet Nam and has played a critical role in responding to the COVID-19 pandemic. In its role as a reference laboratory for the entire country, NIHE received some of the earliest specimens from cases of suspected COVID-19. We describe herein the virological characteristics of specimens received for COVID-19 testing between January and April 2020.

## Methods

Viet Nam established a National Steering Committee on Prevention and Control of COVID-19 on 28 January 2020, 6 days after the first cases of COVID-19 were identified in the country. (*3*) Subsequent guidelines issued by the Steering Committee on 19 February 2020 called for the collection of nasopharyngeal and oropharyngeal (NP/OP) swabs from suspected cases and close contacts of confirmed cases; the guidelines were harmonized with those of the World Health Organization (WHO) in March 2020. (*1*) Additional samples were obtained from travellers in quarantine, who were required to provide upper respiratory specimens for testing upon arrival and before the end of the 14-day quarantine. Specimens were submitted by hospitals, provincial centres for disease control or quarantine facilities, with forms to indicate the reason for testing. Confirmed cases of COVID-19 were sampled every 3 days during hospitalization until they recovered clinically and had at least two negative results by real-time reverse transcription polymerase chain reaction (RT–PCR) for SARS-CoV-2.

### Real-time RT–PCR testing

NP/OP swabs were placed into a viral transport medium and maintained at 4 °C during transport to the national influenza centre at NIHE for 24–48 hours. (*7*) RNA was isolated from the swabs with the viral RNA extraction kit (Qiagen, Hilden, Germany) according to the manufacturer’s instructions in biosafety level 3 containment laboratories. Real-time RT–PCR was conducted with the SuperScript III One-step RT–PCR system with Platinum Taq High Fidelity DNA Polymerase (Invitrogen, Carlsbad, CA, USA), with targets of E, RdRp and N genes according to WHO recommendations. We defined confirmed cases as those with cycle threshold (Ct) values < 37 for at least two of the target genes. (*8*)

### Viral isolation

Vero E6 cells were maintained in Eagle’s minimal essential medium containing 5% (v/v) newborn calf serum; 100 µL of real-time RT–PCR-positive samples were inoculated onto Vero E6 cells and incubated at 37 °C. Viral growth was monitored by daily observation of cytopathic effect. All experiments with SARS-CoV-2 viruses were performed in biosafety level 3 containment laboratories. (*9*)

### Data analysis

Laboratory and epidemiological data collected in this study were entered into a FileMaker Pro 19 Advanced database and analysed. Summary data were reported to the Minister of Health daily and fed back to each sender 24–48 hours after reception of samples.

## Results

### Characteristics of specimens received

Between 23 January and 25 May, the national influenza centre received 15 650 NP/OP specimens from 14 470 suspected cases in 28 cities and provinces in northern Viet Nam. Samples were submitted from two types of suspected cases: 6420 people who entered Viet Nam from abroad (China during the first cluster and other countries during the second cluster) and 8050 from people who were contacts of suspected or confirmed cases. During the first cluster (23 January–25 February 2020), 1741 specimens (11% of all specimens) were collected from people arriving from Wuhan, China, their families and their close contacts. During the second cluster of cases (7 March–25 May 2020), we received an additional  13 909 (88.9%) samples; nearly two thirds were received from four locations (Hanoi, 5366; Ha Giang, 1603; Thai Binh, 1118; Lai Chau, 1011) (**Table 1**).

**Table 1:** Epidemiological features of suspected cases tested for SARS-CoV-2, northern Viet Nam, January–May 2020

Dates	Source of suspected cases	No. of suspected cases	Gender, *n*(%)		Mean (IQR)	No. of positive cases (%)
			Male	Female		
23 January– 25 February	Travellers from China	1123	516 (45.9)	607 (54.1)	30 (1 month–87 years)	6 (0.5)
	Community contacts	118	54 (45.8)	64 (54.2)	35 (4 months–58 years)	7 (5.9)
7 March– 25 May	Travellers from other countries	5297	2436 (45.9)	2861 (54.1)	38 (1 month–96 years)	83 (1.6)
	Community contacts	7932	3648 (46.0)	4284 (54.0)	33 (1 month–90 years)	62 (0.8)
**Total**		14 470	6654 (45.9)	7816 (54.1)	34 (1 month–96 years)	158 (1.1)

### Detection of SARS-CoV-2 by real-time RT–PCR

Among 14 470 tested samples, 158 (1.14%) cases of SARS-CoV-2 were confirmed (**Table 1**). Eighty-nine (56%) of these were detected among suspected cases imported from other countries and the remaining 69 (44%) among community contacts of confirmed cases (**Table 2**).

**Table 2:** Epidemiological features of confirmed cases of COVID-19, northern Viet Nam, January–May 2020

Group	All cases, *n*(%)	Gender, *n*(%)		Nationality, *n*(%)		Mean (IQR)	Re-positive, *n*(%)
		Male	Female	Viet Nam	Others		
Imported cases	89 (56)	45 (51)	44 (49)	74 (83)	15 (17)	33 (10–74 years)	6 (3.8)
Community contacts	69 (44)	17 (25)	52 (75)	69 (100)	0 (0)	41 (3 months–88 years)	1 (0.6)

Thirteen cases were confirmed during the first cluster among people returning from China or their close contacts. Of the 158 confirmed cases, 143 (91%) were Vietnamese nationals and 96 (61%) were female, although we observed a significant difference in the distribution of gender between imported cases (44/89 or 49% female) and cases among community contacts (52/69, or 75% female, *P* < 0.0009 by the χ^2^ test). The median age was 41 years (interquartile range [IQR]: 3 months–88 years) for community contacts and 33 years (IQR: 10–74 years) for imported cases. Eleven (12%) of the 89 imported cases were detected only at second sampling while in quarantine.

The Ministry of Health guidelines require that laboratory-confirmed cases undergo follow-up testing until at least two consecutive tests are negative. Most cases required three or four subsequent tests to meet this criterion, but we also observed some cases after the collection of 10–15 subsequent specimens (**Table 3**).

**Table 3:** Relations between cycle threshold (Ct) values and specimen positivity over time for 158 confirmed cases of COVID-19, northern Viet Nam, February–May 2020

No. of tests for each suspected case	< 30		^3^30		Positive		Negative	Total
	*n*	%	*n*	%	*n*	%	*n*	*n*
1	105	71	42	29	147	93	11^a^	158
2	42	62	26	38	68	53	61	129
3	12	43	16	57	28	23	96	124
4	6	55	5	45	11	15	60	71
5	0	0	4	100	4	8	45	49
6	1	50	1	50	2	5	40	42
7	0	NA	0	0	0	0	31	31
8	0	0	2	100	2	10	19	21
9	1	50	1	50	2	17	10	12
10	0	0	2	100	2	25	6	8
11	0	0	0	0	0	0	3	3
12	0	0	0	0	0	0	1	1
13	0	0	0	0	0	0	1	1
14	0	0	0	0	0	0	1	1
15	0	0	0	0	0	0	1	1
Total	167	63	99	37	266	69	386	652

^a^ The first samples from these cases were negative, but the second samples were positive. All were from travellers from countries other than China.

### Correlation between Ct value, date of illness/days since first positive sample and viral culture results

We analysed the Ct values of 158 confirmed cases of SARS-CoV-2 infection by serial sampling during hospitalization until two consecutive negative results were obtained. The proportion of cases that tested positive decreased with the number of times they were sampled. Among the 652 samples collected, 167 (26%) had Ct values < 30, of which 105 (63%) were identified at the first sampling. Among cases that were sampled a third and fourth time, only 12/124 (10%) and 6/71 (8%) cases, respectively, had Ct values < 30 (**Table 3**).

We identified 99 positive specimens with Ct values > 30, including seven cases that tested positive again after having tested negative (“re-positives”). The pattern was similar to that of cases with Ct values < 30: for 84 (85%) cases, only the first three samples were positive, and an additional 10 (11%) cases had positive results for one of the next three samples. One case was sampled 15 times with no positive results after the 10th sampling.

For 81/158 (51%) confirmed cases, the samples had been appropriately stored and were of a sufficient volume to be inoculated onto Vero E6 cells, from which we obtained 29 (36%) SARS-CoV-2 isolates. Of these, 20 samples had detectable cytopathic effects between 72 and 96 hours, and an additional 9 isolates were harvested after a second blind passage. We identified 28 samples with Ct values < 20, and, of these, 18 (64%) yielded culturable virus (**Table 4**). An additional 20 cases had Ct values of 20–25, and we successfully cultured virus from 10 (50%) of these. The additional nine isolates recovered during the second passage had Ct values of 25–30, suggesting a low load of viable virus. No viral isolates were recovered from samples with Ct values > 30 (*n* = 20).

**Table 4:** Relations between Ct value and culturable SARS-CoV-2 virus, northern Viet Nam, February–May 2020

Ct value	No. of clinical samples	Isolates recovered, *n*(%)
£20	28	18 (64)
21–25	20	10 (50)
26–30	20	1 (5)
> 30	13	0 (0)
Total	81	29 (36)

## Discussion

During the first 5 months of the COVID-19 epidemic in Viet Nam, we characterized all upper respiratory tract specimens received by NIHE from cities and provinces in northern Viet Nam. During that time, two clusters of SARS-CoV-2 infection occurred with community transmission. Just over 1% of all samples yielded positive results by real-time RT–PCR and, by the end of May 2020, fewer than 400 cases had been identified in Viet Nam, with no deaths.

Rapid scaling up and decentralization of testing were key components of Viet Nam’s strategy to minimize entry and transmission of SARS-CoV-2. We identified 89 laboratory-confirmed cases in travellers by testing during centralized quarantine. Of them, 78 (88%) were positive on their first sampling, and 11 were positive during their quarantine. This suggests that testing in quarantine centres at entry and throughout quarantine can prevent transmission of SARS-CoV-2 in a country. Our results provided critical support for evaluating the COVID-19 prevention and control strategy in Viet Nam.

Although viral culture is the gold standard for confirmation of viral infection, real-time RT–PCR is the accepted gold standard for detecting SARS-CoV-2 for the purposes of isolation and contact tracing because of the shorter turnaround time and greater sensitivity. Semi-quantification of viral nucleic acids from the Ct value can be used to select samples for virus isolation. (*3*, *9*-*11*) We observed a strong correlation between Ct values and cell culture positivity rate, suggesting that viral load may be used as a proxy for the infectivity of infected patients.

Among the 158 confirmed COVID-19 cases, seven had positive real-time RT–PCR results after two consecutive negative results within 15 days. Prolonged viral nucleic acid detection in samples from patients who have recovered from COVID-19 has been a concern, as the large majority of these samples, both in the literature and in our collection, have high Ct values, yet attempts to culture these viruses have been unsuccessful. (*4*, *10*) The virus could not be cultured from specimens from the seven cases in this study, all of which had Ct values > 30, suggesting that these cases represent viral remnants rather than infectious virus. These findings are consistent with those from China and the Republic of Korea. (*11*-*14*) This observation supports the hypothesis that prolonged shedding or re-positivity of samples is not associated with continued replication but is rather an indicator of removal of damaged lung tissue containing intact stretches of viral RNA by coughing or ciliary transport. (*13*, *14*) Positive real-time RT–PCR results can be confusing for patients and hospital staff who understandably wish to prevent continued transmission, either among patients and health-care workers or in the general community. These findings should provide reassurance that patients with positive real-time RT–PCR results with Ct values > 30 more than 10 days after onset or first positive result and after having had a negative result are at extremely low risk of transmission. These findings also support a strategy of testing based on signs of clinical recovery, rather than a “test-of-cure” strategy.

This study had several limitations. First, the specimens we received were collected as part of the national strategy for prevention and control of COVID-19 without accompanying systematic clinical metadata, and we were thus unable to stratify asymptomatic, mild and severe cases. Second, we could not systematically assess the possible duration of viral shedding because most of our cases were detected upon arrival, through contact tracing and in quarantine. Thus, sampling times were determined by disease control staff in the field rather than in the context of a rigorously designed study. Third, the specimens for viral isolation were only from the upper respiratory tract. We did not receive any sputum or tracheal aspirate fluids, which might have different characteristics in terms of Ct values or culturable virus.

In summary, we describe here the virology and epidemiology of cases of laboratory-confirmed COVID-19 in northern Viet Nam in two clusters of cases during the first 5 months of the pandemic. Most cases that were laboratory-confirmed were confirmed within the first few samplings. We also determined that most cases that are positive very late in their clinical course are unlikely to represent active infection but, rather, remnants of viral RNA. These results have provided valuable information for improving technical guidelines for molecular testing, viral isolation and clinical management of COVID-19 in Viet Nam.
